# Genomic Characterization of Cyanophage vB_AphaS-CL131 Infecting Filamentous Diazotrophic Cyanobacterium *Aphanizomenon flos-aquae* Reveals Novel Insights into Virus-Bacterium Interactions

**DOI:** 10.1128/AEM.01311-18

**Published:** 2018-12-13

**Authors:** Sigitas Šulčius, Eugenijus Šimoliūnas, Gediminas Alzbutas, Giedrius Gasiūnas, Vykintas Jauniškis, Jolita Kuznecova, Sini Miettinen, Emelie Nilsson, Rolandas Meškys, Elina Roine, Ričardas Paškauskas, Karin Holmfeldt

**Affiliations:** a Laboratory of Algology and Microbial Ecology, Nature Research Centre, Vilnius, Lithuania; b Center for Ecology and Evolution in Microbial Model Systems, Department of Biology and Environmental Science, Linnaeus University, Kalmar, Sweden; c Department of Molecular Microbiology and Biotechnology, Institute of Biochemistry, Vilnius University, Vilnius, Lithuania; d Thermo Fisher Scientific Baltics, Vilnius, Lithuania; e Faculty of Mathematics and Informatics, Vilnius University, Vilnius, Lithuania; f Department of Protein-DNA Interactions, Institute of Biotechnology, Vilnius University, Vilnius, Lithuania; g Protein Structure and Function Programme, Structural Molecular Biology Group, Novo Nordisk Foundation Center for Protein Research, Faculty of Health and Medical Sciences, University of Copenhagen, Copenhagen, Denmark; h Proteomics Unit, Institute of Biotechnology, Helsinki Institute of Life Science, University of Helsinki, Helsinki, Finland; i Molecular and Integrative Biosciences Research Programme, University of Helsinki, Helsinki, Finland; j CasZyme, Vilnius, Lithuania; Michigan State University

**Keywords:** Baltic Sea, brackish environment, phage-encoded CRISPR-Cas, *Siphoviridae*, TA system

## Abstract

While filamentous cyanobacteria play a crucial role in food web dynamics and biogeochemical cycling of many aquatic ecosystems around the globe, the knowledge regarding the phages infecting them is limited. Here, we describe the complete genome of the virulent cyanophage vB_AphaS-CL131 (here, CL 131), a *Siphoviridae* phage that infects the filamentous diazotrophic bloom-forming cyanobacterium Aphanizomenon flos-aquae in the brackish Baltic Sea. CL 131 features a 112,793-bp double-stranded DNA (dsDNA) genome encompassing 149 putative open reading frames (ORFs), of which the majority (86%) lack sequence homology to genes with known functions in other bacteriophages or bacteria. Phylogenetic analysis revealed that CL 131 possibly represents a new evolutionary lineage within the group of cyanophages infecting filamentous cyanobacteria, which form a separate cluster from phages infecting unicellular cyanobacteria. CL 131 encodes a putative type V-U2 CRISPR-Cas system with one spacer (out of 10) targeting a DNA primase pseudogene in a cyanobacterium and a putative type II toxin-antitoxin system, consisting of a GNAT family N-acetyltransferase and a protein of unknown function containing the PRK09726 domain (characteristic of HipB antitoxins). Comparison of CL 131 proteins to reads from Baltic Sea and other available fresh- and brackish-water metagenomes and analysis of CRISPR-Cas arrays in publicly available A. flos-aquae genomes demonstrated that phages similar to CL 131 are present and dynamic in the Baltic Sea and share a common history with their hosts dating back at least several decades. In addition, different CRISPR-Cas systems within individual A. flos-aquae genomes targeted several sequences in the CL 131 genome, including genes related to virion structure and morphogenesis. Altogether, these findings revealed new genomic information for exploring viral diversity and provide a model system for investigation of virus-host interactions in filamentous cyanobacteria.

**IMPORTANCE** The genomic characterization of novel cyanophage vB_AphaS-CL131 and the analysis of its genomic features in the context of other viruses, metagenomic data, and host CRISPR-Cas systems contribute toward a better understanding of aquatic viral diversity and distribution in general and of brackish-water cyanophages infecting filamentous diazotrophic cyanobacteria in the Baltic Sea in particular. The results of this study revealed previously undescribed features of cyanophage genomes (e.g., self-excising intein-containing putative dCTP deaminase and putative cyanophage-encoded CRISPR-Cas and toxin-antitoxin systems) and can therefore be used to predict potential interactions between bloom-forming cyanobacteria and their cyanophages.

## Abstract (precis)

The genomic characterization of novel cyanophage vB_AphaS-CL131 and the analysis of its genomic features in the context of other viruses, metagenomic data, and host CRISPR-Cas systems contribute toward a better understanding of aquatic viral diversity and distribution in general and of brackish-water cyanophages infecting filamentous diazotrophic cyanobacteria in the Baltic Sea in particular. The results of this study revealed previously undescribed features of cyanophage genomes (e.g., self-excising intein-containing putative dCTP deaminase and putative cyanophage-encoded CRISPR-Cas and toxin-antitoxin systems) and can therefore be used to predict potential interactions between bloom-forming cyanobacteria and their cyanophages.

## INTRODUCTION

Cyanophages can exert significant control on cyanobacterial population dynamics, influencing species turnover, diversity, and evolution (1–3) and, thereby, the biogeochemical cycling and other functions of the aquatic ecosystems (4, 5). Although cyanophage research has received considerable attention over the past few decades, the information regarding cyanophage genetic diversity and cyanophage genome contents remain, in the current databases, severely limited and heavily biased toward very few cyanobacterial host species, marine environments, and certain groups of viruses. For example, from 231 publicly available sequenced cyanophage genomes to date (NCBI, accessed on 17 October 2017), 94% belong to viruses infecting the unicellular cyanobacteria *Synechococcus* and *Prochlorococcus* (e.g., 6–12). The majority of large-scale metagenomic surveys covering data from both wide geographical regions and isolated cyanophages deal with marine environments (12–14). In comparison, the freshwater cyanophages are still largely under-represented in the current (meta)genomic databases (15–17).

Even less is known about cyanophages from the largest brackish-water environments, such as the Baltic, Black, and Caspian Seas and their coastal ecosystems (18, 19). Phylogenetic studies have demonstrated that freshwater cyanophages are genetically and evolutionarily distinct from their marine counterparts (20), suggesting that a large proportion of cyanophage diversity remains unexplored. Further, the majority of cyanophages seem to belong to either of two families of *Myoviridae* and *Podoviridae*, sharing conserved genes of structural proteins, and possess genome organizations resembling those of T4-like and T7-like bacteriophages, respectively (11, 12, 21, 22). On the other hand, although globally widespread (23, 24), cyanophages within the family *Siphoviridae* have been shown to be present in significantly lower densities in pelagic ecosystems than myo- and podocyanophages (22). However, most of the siphocyanophage genomes show little resemblance to each other and represent a highly diverse group of viruses compared to their myo- and podocyanophage counterparts (22, 25). The above-mentioned examples together illustrate the uneven distribution of known cyanophage sequences with respect to their hosts, environment, and evolutionary context. Consequently, this hampers the comprehensive understanding of total cyanophage diversity.

Filamentous cyanobacteria are distributed globally and play a crucial role in food web dynamics and biogeochemical cycling in many aquatic ecosystems (26–29). Some species are well known for forming harmful and toxic blooms that are expected to increase in frequency and intensity owing to climate change and anthropogenic pollution (30, 31). Therefore, factors controlling the proliferation and dynamics of filamentous cyanobacteria have been studied extensively. However, the genomic diversity of cyanophages infecting and lysing these cyanobacteria has largely been unexamined. For example, to date there are only 11 sequenced genomes (including the one presented in this study) attributed to cyanophages that infect filamentous cyanobacteria (Table 1). Moreover, these genomes were derived from cyanophages infecting only six different host species (Table 1). The lack of knowledge regarding cyanophage diversity precludes our understanding the genetic potential of the viruses that influence the evolution and ecology of the environmentally relevant cyanobacteria.

**TABLE 1:** Sequenced genomes of bacteriophages infecting filamentous cyanobacteria (as of December 2017)

Cyanophage	Host organism	Capsid size (nm)	Tail length (nm)	Genome size (Kb)	GC%	No. of ORFs	No. of tRNAs	Virus family^*a*^	GenBank accession no.	Reference
vB_AphaS-CL131	Aphanizomenon flos-aquae strain 2012/KM1/D3	97	361	112.8	39.7	149	2	*Siphoviridae*	MG209611	This study
A-4L	*Anabaena* sp. strain PCC 7120	NF^*b*^	NF	41.8	43.4	38	0	*Podoviridae*	NC_024358.1	114
N1	*Nostoc* sp. strain PCC 7120	NF	NF	65.0	35.1	89	0	*Myoviridae*	KU234532.1	97
A1	*Nostoc* sp. strain PCC 7120	NF	NF	68.3	36.5	97	0	*Myoviridae*	KU234533.1	97
vB_NpeS-2AV2	Nodularia spumigena strain UHCC 0040	95	795	139.1	40.3	182	1	*Siphoviridae*	KU230356.1	24
PaV-LD	Planktothrix agardhii strain HAB637	100	NF	95.3	41.4	142	0	*Podoviridae*	NC_016564.1	66
PP	Phormidium foveolarum^*a*^	52	NF	42.5	46.4	41	0	*Podoviridae*	NC_022751.1	115
Pf-WMP3	Phormidium foveolarum^*a*^	55	NF	43.3	46.5	41	0	*Podoviridae*	NC_009551.1	64
Pf-WMP4	Phormidium foveolarum^*a*^	55	NF	40.9	51.8	45	0	*Podoviridae*	NC_008367.1	116
MIS-PhV1A	*Phormidium* sp.	NF	NF	45.5	40.0	62	0	NF	KF437907.1	Unpublished
MIS-PhV1B	*Phormidium* sp.	NF	NF	41.3	40.1	57	0	NF	KF437908.1	Unpublished

a Morpholohgical description was taken from NCBI or inferred from the available literature.

b NF, no information on cyanophage morphology was found in the publicly available literature or NCBI database.

In this paper, we present the genomic analysis of cyanosiphovirus vB_AphaS-CL131 (here, CL 131) (see Fig. S1 in the supplemental material) previously isolated from the Curonian Lagoon (southeastern part of the Baltic Sea), which infects the diazotrophic filamentous cyanobacterium *Aphanizomenon flos-aquae* (strain 2012/KM1/D3) (32), a frequent bloom-forming species in fresh- and brackish-water ecosystems worldwide (33, 34). The results demonstrate that CL 131 has little similarity to previously characterized viruses, suggesting the existence of a distinct and yet uncharacterized phylogenetic lineage. The CL 131 genome encodes a unique set of proteins that have not previously been found in any currently known cyanophages and that potentially contribute to increased cyanophage fitness during infection. This study expands our knowledge of the gene pool harbored by cyanophage in general and reveals the previously undetermined genetic potential of viruses infecting filamentous cyanobacteria in particular.

## RESULTS AND DISCUSSION

### Optimization of read number for the genome assembly.

The relation between the read number used for the assembly, the longest assembled contigs, and the *N*_50_ contig values (the minimum contig length needed to cover 50% of the genome) is given in Fig. S1 in the supplemental material. The analysis indicated that the quality of the assembly was dependent on the number of reads used (Fig. S1). The length of the longest contigs and *N*_50_ readily increased until the read number reached 20,000 and started to decrease when the read number became greater than ∼50,000 (Fig. S1). Based on these observations, the level of 30,000 reads was chosen for further subsampling used in the genome assembly of cyanophage CL 131. A similar approach for subsampling reads in order to facilitate assembly of longer contigs was successfully applied for assembly of viral metagenomes (35). The control of the number of reads was also used for the *de novo* assembly of viral genomes (36) and might be important to prevent the deteriorating effect of the excessive read coverage on the final genome assembly.

### General characteristics of vB_AphaS-CL131 cyanophage genome.

**FIG 1:**
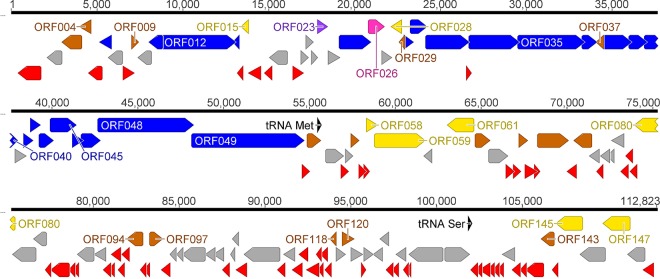
Genome map of cyanophage vB_AphaS-CL131 (CL 131) with annotated ORFs and assigned gene functions as described in the text. The color code is as follows: yellow, DNA replication, recombination, repair, and packaging; brown, transcription, translation, and nucleotide metabolism; blue, structural proteins; purple, chaperones/assembly; pink, protein with predicted catabolic activity; gray, ORFs of unknown function; red, CL 131-specific ORFs that encode unique proteins with no reliable identity to database entries; black, tRNA.

The CL 131 cyanophage has a linear double-stranded DNA (dsDNA) genome 112,793 bp long with an average G+C content of 39.7%, which is close to the average G+C content of the A. flos-aquae host strain 2012/KM1/D3 (37.7%) (37). The results of PCR and restriction digestion analyses (data not shown) suggest that the CL 131 genome is circularly permuted and terminally redundant. Similar to other dsDNA bacteriophages, the coding sequences were relatively closely packed, with some overlapping genes, and occupied almost 91% of the nucleotide sequence (Fig. 1). Most of the open reading frames (ORFs) were found to initiate with ATG (86%), yet several ORFs started with GTG (7%) and TTG (7%) as the initiation codon (Table S2).

Two tRNA genes (tRNA^Ser^ and tRNA^Met^) and 149 protein-coding genes were predicted in total (Table S2), of which 56 genes are on the plus strand and 93 genes are on the minus strand (Fig. 1 and Table S2). The relatively small number of tRNA genes found in CL 131, the lack of RNA polymerase (Table S2), and a codon usage pattern similar to that of the host (data not shown) suggest that this virus has specialized toward infecting A. flos-aquae host strain 2012/KM1/D3 and explicitly relies on the cyanobacterial transcription and translation machinery (38). The low number of tRNA genes might also be related to a relatively narrow host range (39), which was observed for this cyanophage. CL 131 was able to infect only 2 out of a total of 60 tested strains belonging to two closely phylogenetically related genera, *Aphanizomenon* and *Dolichospermum* (40). Both infection-sensitive A. flos-aquae strains were isolated from the Curonian Lagoon (Table S1). CL 131 failed to infect cyanobacterial strains isolated from other fresh- and brackish-water ecosystems, including the Baltic Sea and lakes in Germany, Lithuania, and Poland (Table S1). Further, the low number of tRNA genes might negatively affect cyanophage fitness (e.g., latency period and replicative capacity [burst size]) (41).

In total, 21 σ^70^-like promoter sequences and 15 ρ-independent transcriptional terminators were predicted in the CL 131 genome (Table S3). The putative CL 131 promoters lacked highly conserved sequences/motifs observed in bacterial (e.g., Escherichia coli) promoters within the −10 and −35 regions (TTGACA and TATAAT, respectively). Predicted promoter sequences exhibited higher variability in the consensus sequence (Fig. S2) than those of myo- and podocyanophages (e.g., Pf-WMP4, S-PM2, N1, and S-CRM01) (23, 42–44) while being more similar to the sequence of siphocyanophage P-SS2 (25). No phage-like promoters were predicted in the intergenic regions of the CL 131 genome (Table S3).

### Phylogenetic analysis of cyanophage vB_AphaS-CL131.

**FIG 2:**
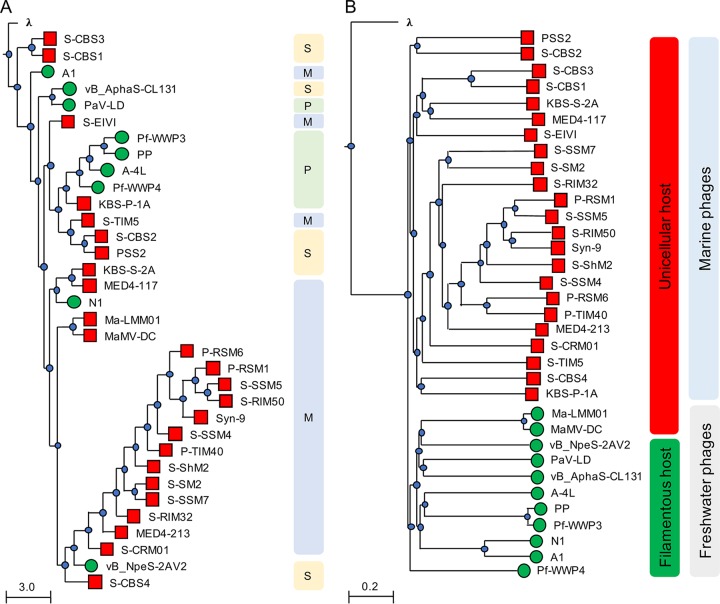
Phylogenetic analysis of cyanophage terminase large (TerL) subunit (A) and nucleotide sequence-based whole-genome comparisons (B) as tree diagrams. The scale bars indicate the average number of amino acid or nucleotide substitutions per site. Bacteriophage λ (family *Siphoviridae*) sequences were used as an outgroup. Red squares and green circles refer to cyanophages infecting unicellular and filamentous cyanobacteria, respectively. Bacteriophage family (M, *Myoviridae*; P, *Podoviridae*; S, *Siphoviridae*) assignments (A) or host cellular arrangement types (red, unicellular; green, filamentous) and habitat (marine or freshwater) (B) are provided.

**FIG 3:**
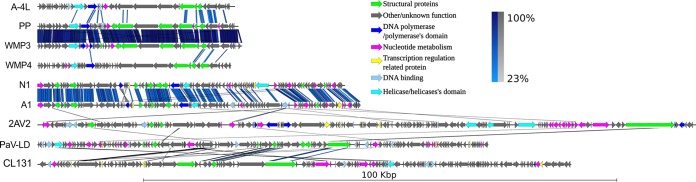
Multiple pairwise genome alignments of cyanophages infecting filamentous cyanobacteria. The scale bar indicates the genome length. Blue and gray bars correspond to normal and inverted BLAST matches, respectively. WMP, Pf-WMP3; WMP4, Pf-WMP3; 2AV2, vB_NpeS-2AV2; CL131, vB_AphaS-CL131.

A phylogenetic analysis based on the inferred amino acid sequences of the terminase large subunit (TerL; ORF059), a universally present conserved phage structural protein, revealed that although CL 131 branched most closely with *Planktothrix* phage PaV-LD, TerL clustered distinctly from TerL proteins of other cyanophages (Fig. 2A). The whole-genome nucleotide sequence alignments of cyanophages infecting unicellular and filamentous cyanobacteria provided further evidence for the evolutionary divergence of the analyzed genomes (Fig. 2B). First, in agreement with a previous study (20), we found that phages infecting freshwater cyanobacteria clustered separately from phages infecting the marine cyanobacteria *Synechococcus* and *Prochlorococcus* (Fig. 2B). Second, cyanophages within the clade of phages that infect freshwater cyanobacteria, including CL 131, were mainly distantly related to each other, whether they infected filamentous or single-celled hosts (Fig. 2B). These observations suggested that viruses infecting filamentous cyanobacteria might have evolved independently both from each other and from those infecting unicellular cyanobacteria. The subsequent genome-wide protein-based comparisons demonstrated a low degree of relatedness and synteny (gene order) (Fig. 3) between cyanophages belonging to the same viral families (e.g., between PaV-LD, A-4L, Pf-WMP3, and Pf-WMP4 or vB_NpeS-2AV2 and vB_AphaS-CL131). This implied a high genomic divergence within this group of viruses compared to divergence of those cyanophages infecting unicellular cyanobacteria such as *Synechococcus*/*Prochlorococcus* (11). Taking the above-mentioned results together, we suggest that CL 131 as well as some other filamentous cyanobacterium-infecting viruses (Fig. 2 and 3) represent novel and previously unrecognized branches of viruses. In comparison to other relatively well-studied cyanophage-cyanobacterium systems (e.g., viruses infecting *Synechococcus* or *Prochlorococcus*), these novel genomes open new avenues for the further phylogenomic studies of cyanophages to better understand their diversity and diversification in aquatic ecosystems.

### Genome content analysis of cyanophage vB_AphaS-CL131.

The BLASTp analysis revealed that more than half (85 out of 149) of identified ORFs have recognizable homologs with proteins found in other bacteriophages (41 ORFs), cyanobacteria (38 ORFs), bacteria (5 ORFs), and archaea (1 ORF) (Table S2). However, only 25% of these ORFs (21 out of 85) can be assigned to function based on sequence homology (Table S2). Among the CL 131 gene products with detectable homologs in other genomes of cyanophages (retrieved using a BLASTp search against the NCBI viral database), the largest number of homologs were similar to the proteins of *Planktothrix* phage PaV-LD (23 out of 36) and Nodularia spumigena cyanophage vB_NpeS-2AV2 (12 out of 36), and these were mainly associated with CL 131 structural proteins (Tables S2 and S4; see also below). Homologs were also found among cyanophages that infect the unicellular cyanobacteria *Synechococcus* and *Prochlorococcu*s, where the majority of matches were to CL 131 ORF036 (unidentified structural protein) and ORF051 (*purM*) (Table S4). A large number of ORFs (64, or 43% of the total number of ORFs) were considered to be hypothetical proteins apparently unique to this phage as they possessed no homologs in the NCBI nonredundant (nr) database. This points toward the underrepresentation of fresh- and brackish-water cyanosiphoviruses infecting filamentous cyanobacteria in the current microbial genome databases.

### Structural proteins, virion morphogenesis, and DNA packaging.

A comparative analysis of the genome sequence of CL 131 allowed the identification of terminase small and large subunits (encoded by ORF058 and ORF59, respectively), which are essential for phage DNA packaging, as well as of four structural and virion morphogenesis-related genes, including those coding for portal (ORF012), major capsid (ORF040), tail tape measure (ORF049), and phage virion morphogenesis (ORF023) proteins (Tables S2 and S4). On the other hand, bioinformatics approaches failed to identify genes encoding essential conserved structural proteins, including major tail protein, baseplate components, or tail fiber proteins, which, based on the results of transmission electron microscopy (TEM) analysis (Fig. S3), are likely to be present in the genome of cyanophage CL 131.

In order to detect unique structural components of CL 131 virions, which have no reliable identity to the database entries, liquid chromatography-tandem mass spectrometry (LC-MS/MS) analysis of purified CL 131 particles was performed. It led to the identification of 23 structural proteins in total, including 19 with previously unknown functions as well as the four structural proteins mentioned above, which were identified by bioinformatics analysis (Table S5 and Fig. S4). Of these 23 CL 131 structural proteins identified by LC-MS/MS analysis, 16 showed similarity with proteins from cyanophages (Table S4). This includes *Planktothrix* phage PaV-LD (13 homologous structural proteins) (Table S4), which is currently classified as a member of the family *Podoviridae* even though it was initially was characterized as a tailless cyanophage (45). Such a relatively high number of homologs of structural genes shared between these two morphologically different cyanophages hints at their common evolutionary history. The same observation has been reported before for podo- and siphophages infecting Escherichia coli (46–48), once again pointing out the known taxonomic anomaly when either genetic or morphological criteria are used. Identification of structural proteins (using LC-MS/MS) that previously were predicted as hypotheticals in other cyanophages (Table S4) provides important information for subsequent studies of new cyanophages as well as for metagenomics.

Finally, although the overall genome architecture of CL 131 is comparable to that of other members within the *Siphoviridae* family, where structural proteins cluster together with genes involved in virion morphogenesis and DNA packaging (Fig. 1; Tables S2 and S5), the gene organization within this cluster deviates from the conserved order described in other tailed bacteriophages and members of the family *Siphoviridae* (49). For example, ORFs encoding CL 131 terminase (ORF058 and ORF059) and portal (ORF012) proteins are located on the opposite sides of the region of the structural genes. Thus, these results broaden our view of the genome structures of viruses infecting cyanobacteria.

### DNA replication and nucleotide metabolism genes.

In total, six ORFs (ORF028, ORF051, ORF069, ORF080, ORF120, and ORF145) were found to be associated with cyanophage CL 131 nucleotide metabolism and DNA replication (Table S2). The ORF080 was predicted to encode SF4 DNA helicase and contains a conserved DnaB domain. Therefore, this protein might be involved in unwinding of DNA duplex, loading of DnaC, and recruitment of other proteins (DnaA, DnaI, PriA, DnaG, etc.) that are necessary to ensure proper regulation of cyanophage DNA replication initiation (50). It has also been shown that DnaB interacts with DnaD, the motif of which was found in ORF132 (Table S2), to recruit the DNA primases and initiate primosome assembly. DnaD was also shown to have DNA-remodelling activity and, together with DnaB and DnaI, to play an important role in control and modulation of bacterial nucleoid architecture (51). CL 131 also encodes DNA polymerase III subunit beta (OFR145) and endodeoxyribonuclease RusA (ORF028). The latter belongs to protein family of Holliday junction resolvases and is suggested to take part in DNA repair during phage replication. The ORF120 was identified as polynucleotide kinase/phosphatase (PKN), which belongs to a broad family of 5′ kinase/3′ phosphatases, enzymes with nucleic acid-modifying activities (52). It has been suggested that PKN helps to establish the required intracellular DNA structure.

The other two identified ORFs were homologs to phosphoribosylaminoimidazole synthetase (PurM; ORF051), an enzyme involved in purine ribonucleotide biosynthesis, and dCTP deaminase (ORF069), an enzyme that supplies deoxyuridine monophosphates to thymidylate synthetase and is thought to regulate the levels of dNTP required for phage DNA synthesis (53). Both of these genes are thought to be auxiliary metabolic genes (AMGs), expression of which is generally assumed to increase phage fitness (54). Among the sequenced cyanophage genomes, the *purM* genes having matches to CL 131 can be commonly found in cyanophages infecting *Synechococcus* and *Prochlorococcus* cyanobacteria, while dCTP deaminase is less frequently observed (Table S6). The dCTP deaminases have also been reported in other cyanophages resembling the morphology of the *Siphoviridae* family (e.g., S-CBS1, S-CBS4, P-SS2, and vB_NpeS-2AV2) (22, 24, 25). The CL 131 dCTP deaminase (ORF069) contains an intein, which we show here experimentally (see section below) can self-excise after expression in E. coli (Fig. S5). Other genes involved in DNA replication (e.g., DNA primase, gyrase, DNA-binding proteins, DNA ligase, etc.) were either missing in the CL 131 genome or lacked recognizable homologs in current databases. The lack of other identifiable proteins involved in nucleotide metabolism and DNA replication suggests that CL 131 recruits host proteins to ensure cyanophage replication, which is a well-known strategy among phages (55–57).

### Characterization of dCTP deaminase.

Bioinformatic analysis revealed that cyanophage CL 131 ORF069 encodes predicted dCTP deaminase with an inserted HNH endonuclease. Based on the results of BLASTp analysis, the N-terminal (amino acids [aa] 1 to 132 aa) and C-terminal (aa 484 to 555) domains of ORF069 form a protein which shares the highest identity (68%) at amino acid level with dCTP deaminase from Calothrix elsteri; an inserted fragment (aa 133 to 483) contains conserved HintN (aa 132 to 223) and HNH_3 (aa 259 to 304) domains. Similarly, HHpred analysis showed that amino acids 1 to 132 and 484 to 555 of ORF069 are predicted to adopt the fold of the dCTP deaminase from Burkholderia thailandensis (PDB accession number 4DHK) with a probability of 100.0 (E value, 2E−33). The fragment from aa 133 to 483 of ORF069 is aligned with the intein homing endonuclease II from Thermococcus kodakarensis (PDB accession number 2CW8) with a probability of 99.12 (E value, 7E−11).

The activity of the intein homing endonuclease within ORF069 of CL 131 was demonstrated experimentally using protein expression vectors. SDS-PAGE analysis revealed that the molecular weight (about 25 kDa) of purified gp069_C-his (Fig. S5) corresponds to the predicted molecular weight (23.8 kDa) of only the gp069 dCTP deaminase coding fragment (aa 1 to 132 and aa 484 to 555).

Intein-containing proteins have previously been found in different bacteriophages (58, 59) and have been shown to be associated with proteins involved in DNA metabolism (60). The HNH endonucleases were also suggested to play a role in the bacteriophage life cycle, fitness, and DNA packaging as well as in the response to environmental stress conditions (58, 61–66). Reports on the self-splicing intron-containing dCTP deaminase are less common (65), and these proteins have only been found so far in two bacteria belonging to the phyla *Proteobacteria* and *Chloroflexi* (67). However, the location of ORF069 within the CL 131 genome makes it difficult to predict the potential role of HNH endonuclease as most of the ORFs next to dCTP deaminase lack a predicted function (Table S2).

### Cyanophage-encoded CRISPR-Cas system.

**FIG 4:**
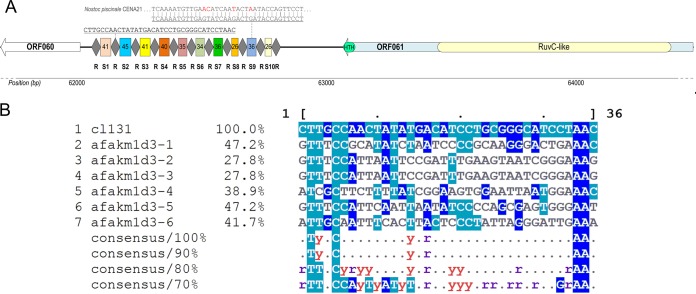
Representation of CRISPR-Cas locus in cyanophage vB_AphaS-CL131 genome (A) and multiple alignments of direct repeats of the cyanophage (CL 131) and Aphanizomenon flos-aquae strain 2012/KM1/D3 (afakm1d3-1 to afakm1d3-6) (B). Alignments were created using the CL 131 direct repeat sequence as the reference sequence; identities are normalized by aligned length and colored by identity. R, direct repeat; S1 to S10, spacers.

Cyanophage CL 131 encodes a putative CRISPR-Cas locus (bp 62058 to 62813) containing 10 spacers of various lengths (26 to 45 nucleotides [nt]) (Table S7) and direct repeats 36 nt long (Fig. 4A). BLASTn analysis showed that only one spacer had a significant (E value of 5E−03) match in the nonredundant NCBI database, partially matching (4 mismatches) a DNA primase pseudogene in cyanobacterium Nostoc piscinale strain CENA21 (NCBI accession number CP012036.1) (Fig. 4A). The direct repeat sequences were well conserved, with only two repeats having a C-to-T mutation, which reflects the stability of this region, which, in turn, is necessary for CRISPR functionality (68). Adjacent to the CRISPR array, a transposon-encoded TnpB protein was identified (ORF061) (Table S2). This protein contains a C-terminal HTH motif and an N-terminal RuvC-like nuclease domain (Fig. 4A), homologous to the large type V effectors Cas12a and Cas12b (69, 70). Comparisons of CL 131 transposon-encoded TnpB with recently reported novel CRISPR-Cas effector proteins (70) suggest that this CL 131-encoded system could be assigned to the putative type V-uncharacterized (V-U) subtype. Further predictions using CRISPRdisco (71) and sequence alignments of CL 131 TnpB with the type V-U2 effector proteins (Fig. S6) confirmed that cyanophage-encoded CRISPR-Cas belongs to the type V-U2 CRISPR-Cas systems (70). The type V-U2 effector proteins are highly prevalent in cyanobacteria compared to their frequency in other phylogenetic groups (70).

It has been proposed, however, that type V-U effectors, including type V-U2, are too small to form a complex sufficient to carry out CRISPR RNA (crRNA)-dependent DNA cleavage (70) as one would expect in type V CRISPR-Cas systems. Regarding the whole CL 131 CRISPR-Cas locus, other Cas protein-related or transacting small RNA (tracrRNA) sequences (71) were absent around the CRISPR-Cas region, leaving the locus architecture minimal. This may suggest that the cyanophage CRISPR-Cas system has a gene-regulatory role (70). The directionality of the CRISPR array remains elusive although one of the predicted repeat secondary structures resembles that observed in type V-A CRISPR-Cas systems (Fig. S7). A hairpin with a 5-bp stem and 6-nt loop could be formed (Fig. S7), suggesting that the manner of binding of the crRNA 5′ handle to the effector protein is similar to that in type V-A systems. However, further analysis is required to determine the function and mode of action of cyanophage-encoded CRISPR-Cas system.

Cases have been recorded in which viruses inherit elements of CRISPR-Cas systems from their bacterial hosts (72) by horizontal gene transfer (HGT) (73, 74). In spite of that, the origin of the identified CRISPR-Cas locus in the CL 131 genome remains unclear. While the identical G+C content (37.3%) of the cyanophage CL 131 CRISPR array and the host A. flos-aquae strain 2012/KM1/D3 genome (37) potentially links the origin of the CL 131 CRISPR array to the host, other results point to HGT as an unlikely event for recruitment of the CRISPR array found in CL 131 from the host. First, the host strain (A. flos-aquae strain 2012/KM1/D3) and other A. flos-aquae strains whose genomes are available today (for details, see reference 40) possess CRISPR systems belonging to type III-B and I-D (class 1) (69), which differ from the system found in CL 131. Second, the sequence alignment of direct repeats (including reverse complement sequences) shows little similarity between the host and CL 131 (Fig. 4B), again suggesting that the cyanophage-encoded CRISPR array is not a result of HGT from A. flos-aquae strain 2012/KM1/D3. Alternatively, the CRISPR-Cas locus in CL 131 could have been gained from another host existing within the same habitat as A. flos-aquae strain 2012/KM1/D3 but not available as a cultured isolate.

Thus far, only one example of a full bacteriophage CRISPR-Cas system has been described in the literature (75) which counters the host phage-inhibitory island and therefore evades innate immunity. More recently, Chénard and colleagues (76) have reported a CRISPR array found in the genome of cyanophage N1 infecting *Nostoc* sp. strain PCC 7210. Although the authors did not identify any *cas* genes in the N1 genome, the N1 CRISPR array was shown to be transcribed during infection (76). This suggested that N1 either uses the host’s Cas proteins or that unidentified *cas* genes are present in the genome (76). Therefore, we reanalyzed the genome of N1 cyanophage using the latest available information on CRISPR classification and *cas* genes (70, 71) and compared it to that of the CL 131 CRISPR-Cas system. Our observations are in agreement with those of the previous study (76) as no *cas* or *cas*-related genes were found in the N1 genome (Table S8). The comparison of the flanking sequences of the CRISPR loci between two cyanophages (CL 131 and N1) showed no similarity in gene content and order in the corresponding regions of the two cyanophages.

### Cyanophage-encoded TA system.

A pair of genes (ORF066 and ORF067) (Table S9) in the CL 131 genome was predicted as a type II toxin-antitoxin (TA) system by the TAfinder software (77). TA systems usually consist of two genes located next to each other in the same operon, with one gene product acting as a toxin while the other antagonizes the action of the toxin (78). In CL 131, OFR066 is predicted to be N-acetyltransferase (Table S2), which belongs to the GCN5-related N-acetyltransferase (GNAT) superfamily of proteins that are involved in a variety of cellular processes and their regulation (79, 80). It has been recently shown that GNAT family enzymes may constitute a part of TA systems and act as either toxin (81) or antitoxin (82) through different RNA modification activities leading to inhibition of DNA transcription and translation (79, 80).

ORF066 was paired with ORF067, which, as predicted by TAfinder, contain the PRK09726 domain (Table S9). This conserved domain is found in HipB family antitoxins of HipAB TA systems, which in turn are involved in a stress response to a variety of factors by induction of a dormancy state in the cells (83). HipA acts as a toxin, which inhibits protein translation, leading to cell growth arrest and persistence, yet the induction of the dormancy itself is determined by the levels of HipB within the cell (84). However, further examination of ORF067 using the NCBI Conserved Domains Database (CDD) failed to prove the existence of any conserved sequences within this protein. In addition, the CL 131-encoded ORF067 is twice as long (172 aa) as the E. coli HipB protein (88 aa), and although, similarly to HipB, it possesses a DNA-binding domain (SHOCT domain; pfam09851) (85), the sequence alignments indicated little similarity between E. coli HipB proteins (GenBank accession numbers NP_416025.1, Q8FHF3.1, P23873.1, KFB93749.1, and KDF68316.1) and ORF067 (Fig. S8). Therefore, additional tests are needed to demonstrate whether ORF067 can act as an HipB-like antitoxin and how it interacts with the putative host’s (A. flos-aquae strain 2012/KM1/D3) HipAB system (37).

Further analysis of the genomes of other cyanophages infecting filamentous cyanobacteria (Table 1) have demonstrated that TA systems or TA-related proteins are present in some other cyanophages as well (Table S9). For example, *Planktothrix* phage PaV-LD contains an HicAB system, which has also been implicated in formation of persister cells under stress conditions (86). Taking into account that dormancy can be used as a host strategy to prevent viral replication in the infected cells (87), these observations raise an intriguing hypothesis that cyanophage-encoded TA systems may play a role in preventing (e.g., through the expression of antitoxins HipB and HicB) host cells from entering the dormant state and, thus, ensuring continuous and successful cyanophage replication.

### Potential for lysogenic lifestyle.

It is common among bacteriophages belonging to the family *Siphoviridae* to be able to integrate into the host genome as a so-called provirus. CL 131 acts as a virulent cyanophage under the standard laboratory growth conditions (32) and, with the exception of three putative prophage antirepressor homologs (ORF002, ORF062, and ORF094), we were not able to identify any of the known genes associated with a lysogenic lifestyle. The HHpred results indicate that ORF002 contains the COG3561 domain, which belongs to the cl01430 family of AntA/AntB type of prophage antirepressors. This protein also shows similarity (Table S2) to a putative integrase/resolvase of the vB_NpeS-2AV2 cyanophage infecting the filamentous cyanobacterium Nodularia spumigena (24). The ORF062-encoded product has similarity to the COG3617 group, which also encompasses the prophage antirepressor proteins, and belongs to the Bro-N superfamily (cl10591). ORF094 contains two domains, the phage regulatory protein Rha (COG3646) and KilAC (COG3645), both of which are often found in prophage genomes. In addition, the BLASTp analysis revealed that ORF015 has similarity (41%; E value of 6E−18) to the integration host factor subunit beta (IHFB) and contains the IHFB domain cd13836 (belonging to the HU_IHF superfamily, cl00257), which is important for phage site-specific recombination. However, other genes encoding proteins related to lysogenic life style (e.g., integrase, excisionase, repressors, etc.) were absent, suggesting the possibility that CL 131 was once a temperate phage that has lost most of its lysogeny-related genes over time. In addition, no intact prophages or any other phage-related sequences were found in the host genome of A. flos-aquae strain 2012/KM1/D3 (74), suggesting both that lytic infections are the dominant type of interactions within this cyanophage-cyanobacterium pair and that the cyanophage effect on A. flos-aquae evolution through specialized transduction events may be negligible.

### Prevalence of vB_AphaS-CL131-like cyanophages in the Baltic Sea.

**FIG 5:**
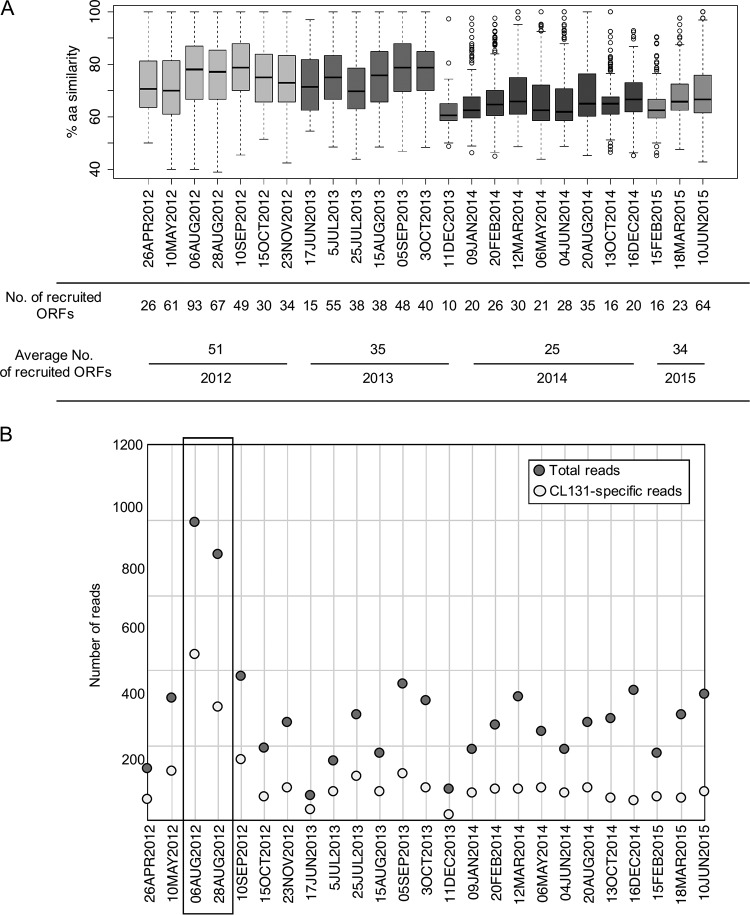
(A) Box plot representing the percentage of amino acid identities shared between cyanophage vB_AphaS-CL131 translated protein sequences and reads of 25 Baltic Sea metagenomes. The box depicts the upper and lower quartiles; the horizontal line indicates the median; whiskers indicate minimum and maximum amino acid identity values; circles indicate outliers. Boxes are colored to represent different years. (B) Dot plot of the number of total and cyanophage vB_AphaS-CL131-specific reads of structural genes. The samples (from August 2012; see text for details) with the highest number of total and vB_AphaS-CL131-specific reads are boxed.

The distribution of CL 131-like cyanophages in different brackish and freshwater environments, in which A. flos-aquae may occur and occasionally form blooms, was assessed by comparing predicted CL 131 proteins to results of metagenomic reads. While matches to only 7% to 20% of CL 131 proteins were detected in the Lake Michigan (United States), Lake Matoaka (United States), and Chesapeake Bay (United States) metagenomes (Table S10), up to 66% of CL 131 proteins had significant matches to the Baltic Sea metagenomic reads (Table S10), which were used for further investigation. After comparing the reads matching CL 131 proteins to proteins in the NCBI database, it was clear that while the majority of reads likely showed spurious matches to conserved regions and had higher bit scores to other viruses or organisms in the NCBI nr database, two time points stand out (Fig. 5).

At both sampling times in August 2012, 45 and 52% of the metagenomic reads either exclusively matched or had higher bit scores to CL 131 proteins than anything else in the NCBI nr database. In addition, these reads matched 45 and 62% of the CL 131 proteins at high amino acid identity (Fig. 5A). These proteins represent a range of functionalities from conserved proteins (e.g., involved in DNA replication) which could be shared by phages belonging to different genera (88) to more phage group-specific proteins (e.g., structural proteins) and proteins thus far unique to CL 131 (Table S2). While it is difficult to determine the presence and importance of CL 131 phage in the Baltic Sea based on these results, our findings indicate that phages similar to and potentially of the same genus (sharing >40% of their genes) (89) as CL 131 were occurring in the Baltic Sea during the late summer of 2012 (Fig. 5B).

Following the summer months in 2012, the number of recruited reads, the number of proteins the reads matched, and the amino acid identity of the matches showed a decreasing trend, with potential increases reoccurring during summer months (Fig. 5), a period when blooms of A. flos-aquae are most likely to happen. However, the findings point only toward distant relations to CL 131 and may suggest an ongoing divergence of CL 131-like viruses in the Baltic Sea over this time period. Given the limited host range detected for CL 131 (Table S10), which agrees with the ranges of other siphocyanophages (6, 90), and the known mosaic genomes of siphoviruses in particular (47, 91), this divergence may not be surprising as the presence of different host strains might promote the presence of different, yet related, phage types. Metagenomic data from other aquatic environments in which A. flos-aquae is prevalent (34) are lacking, thus limiting more comprehensive analysis of global distribution of CL 131-like cyanophages.

As an alternative strategy to reveal the distribution of CL 131-like cyanophages, we analyzed the CRISPR arrays of all publicly available A. flos-aquae genomes to assess the presence of CL 131-like spacers. A BLAST search using all observed cyanobacterial CRISPR spacers in A. flos-aquae genomes revealed hits only in A. flos-aquae strains 2012/KM1/D3 (6 spacers) (Table S11), which is the original host strain for this cyanophage, and NIES-81 (5 spacers) (Table S11), isolated in 1978 from Lake Kasumigaura in Japan. The latter observation suggests that distribution of CL 131-like cyanophages is not restricted only to the Baltic Sea but that these phages might be found in other environments, where they share common history with their host strains dating back several decades.

The observed similarity between cyanobacterium spacer sequences and corresponding sequences in the CL 131 genome vary between 93% and 100% (Table S11), which implies an ongoing coevolution between virus and cyanobacterial species and that CL 131 cyanophages continue to exert pressure and shape the evolution of A. flos-aquae. The cyanobacterial CRISPR spacers in 2012/KM1/D3 and NIES-81 strains matching the CL 131 genome target both less-conserved structural and morphogenesis genes (e.g., ORF012, ORF023, and ORF040) (Table S11) and highly conserved phage genes (e.g., DNA helicase, ORF080) that are crucial for phage replication and survival. Further, the spacers against the CL 131 cyanophage were found in multiple CRISPR arrays in both strains (Table S11), indicating that several different CRISPR-Cas systems within the same cyanobacterium might target the same phage. Finally, we also found that two different A. flos-aquae strains target the same type of cyanophage genes (structural and morphogenesis genes).

### Concluding remarks.

Overall, this study provides insight into the genomic characteristics and distribution of a newly isolated virulent cyanophage that infects the bloom-forming filamentous cyanobacterium Aphanizomenon flos-aquae. The high divergence of cyanophages within the well-separated cluster of phages infecting filamentous cyanobacteria, where CL 131 represents an addition for a new lineage of cyanophages, points toward the importance of the characterization of more phage isolates. Our characterization of this divergent cyanophage, including thorough experimental identification of otherwise nonannotatable virion structural proteins, provides a valuable resource for discovery of related genes in the “viral dark matter” common in viral meta-omics studies. The presence of a self-excising intein-containing protein (dCTP deaminase), a CL 131 unique TA system, and a CRISPR-Cas system, even though its functionality still needs to be verified, demonstrate that the genetic pool of environmental phages still provides novel and unusual functionalities. Analysis of the Baltic Sea metagenomes and cyanobacterial CRISPR-Cas systems suggests that although cyanophage CL 131 has a relatively narrow host range, these phages are a dynamic component of the microbial food web actively interacting with their host strains. Given the abundance of A. flos-aquae in the Baltic Sea and other aquatic environments (34) and its crucial role in the food web structure and function, especially during the summer-autumn seasons (92, 93), the genome sequence analysis of cyanophages that infect these cyanobacteria provides valuable insights into the potential of these biotic factors to control and shape the population dynamics and evolution of harmful cyanobacteria.

## MATERIALS AND METHODS

### DNA extraction and sequencing.

Cyanophage DNA was extracted from 1 ml of CsCl_2_-purified cyanophage suspension (∼10^10^ PFU ml^−1^) (32) using the phenol-chloroform extraction and ethanol precipitation method. Isolated cyanophage DNA was subsequently used for restriction analysis, PCR, and genome sequencing. The cyanophage genome was sequenced to ∼10,000-fold coverage using an Illumina MiSeq platform, with a 150-bp paired-end library constructed using a MiSeq reagent kit (version 2).

### Genome assembly of cyanophage vB_AphaS-CL131.

Raw reads were quality checked using FastQC version 0.11.4 (94), and assembled with SPAdes genome assembler (version 3.7.0) (95) with “careful” and all other options set to their default options. Prior to selection of optimum read number for the genome assembly, errors in reads were corrected with SPAdes using the “only-error-correction” flag. The search for optimum read number was performed by testing different subsampling levels ranging from 5,000 to 100,000 reads with a step of 5,000 reads. The qualities of the assemblies were assessed using QUATS version 4.6.0 (96). At the chosen subsampling level (see Results) 100 repeated assemblies were conducted. The resulting set of contigs was filtered by removing contigs with coverage lower than 9 (∼10% below maximum). The remaining contigs were reassembled with SPAdes, resulting in one continuous sequence.

### Genome annotation and analysis.

Genome annotation was performed using DNA Master (version 5.22.23) and Geneious Pro (version 5.5.6.) (45, 97). The translated open reading frames (ORFs) were inspected manually based on prediction of coding potential generated with GeneMark (98) or Glimmer (99) and used as queries to search for sequence homologs by BLASTp (BLAST+ version 2.7.1) in the nonredundant NCBI (NCBI nr; accessed in October 2017) and protein database from viral genomes in NCBI RefSeq databases (accessed in January 2018) with an upper-threshold E value of 10^−5^. For functional annotation, NCBI’s Conserved Domain Database and HHpred (https://toolkit.tuebingen.mpg.de/#/tools/hhpred) (E value of 10^−3^) were used in addition to the NCBI nr database. Genome organization was visualized using Geneious Pro (version 5.5.6.) software. Identification of tRNA genes was performed with tRNAscan-SE (100). Potential promoters were predicted by searching intergenic regions in 150 bp upstream of predicted ORF starts. Putative phage promoter sites were searched with PHIRE using default parameters (101). Putative bacterial σ^70^ promoters were identified with BPROM (SoftBerry, Inc., USA), using a linear discriminant function (LDF) value of >5. Identification of ρ-independent transcriptional terminators was performed with the FINDTERM prediction program (Softberry, Inc., USA) using an energy threshold score of −16 kCal or better. Consensus sequences of the regulatory motifs were created with WebLogo-3 (102).

Phylogeny analysis of the terminase large subunit (TerL) protein sequences was performed using Phyrn (version 1.7.2) (103) with 5,000 replicates of the distance matrices for bootstrapping. Whole-genome, nucleic acid-based phylogenetic analysis was performed using the VICTOR online tool (http://ggdc.dsmz.de/victor.php) with settings (protocol *d*_0_) recommended for prokaryotic viruses (accessed June 2017) (104) and, subsequently, visualized with ETE (version 2.2) (105). The corresponding neighbor-joining trees were calculated, and a consensus tree was produced using the NEIGHBOR and CONSENS programs from PHYLIP (version 3.2).

For pairwise comparisons, cyanophages infecting filamentous cyanobacteria were used (Table 1). Before the pairwise alignments, all cyanophage genomes were reannotated using Prokka (version 1.12) using an E value cutoff of 0.01 (106). The identified protein sequences were further annotated with PANNZER and/or PANNZER2 (107) using the query coverage cutoff value of 0.6, followed by Gene Ontology (GO) number assignment to the annotated proteins. The annotated proteins were further classified into six functional groups: (i) “structural proteins” if the corresponding description contained any of the words tape, structural, baseplate, tail, or capsid or if the term GO:0019028 was assigned; (ii) “DNA polymerase” if the corresponding description contained the phrase DNA polymerase; (iii) “DNA helicase” if the corresponding description contained the term helicase; (iv) “nucleotide metabolism” if the protein was not classified to any of the above-indicated groups and/or the corresponding GO term was one of the following: GO:0009117, GO:0090304, GO:0006259, GO:0090305, GO:0016070, or GO:0006139; (v) “transcription regulation” if the corresponding description contained the phrase tTranscription regulator or if the term GO:0019028 was assigned; and (vi) “DNA binding” if the corresponding description contained the phrase DNA binding or if the term GO:0043565 or GO:0003677 was assigned. The pairwise genome comparisons and visualization of the assigned functional groups were done using Easyfig (108).

### Analysis of the dCTP deaminase gene.

Bioinformatics analysis of ORF069 was performed using Fasta-Nucleotide, Fasta-Protein, BLASTp, Transeq (www.ebi.ac.uk/Tools/st/emboss_transeq), Clustal Omega (www.ebi.ac.uk/Tools/msa/clustalo), and Sequence Editor (www.fr33.net/seqedit.php) software. Molecular weight of the recombinant protein was predicted using the molecular weight calculation tool available at the Protein Information Resource (PIR) database (pir.georgetown.edu/pirwww/search/comp_mw.shtml). Search for the ORF069 fold was performed using the HHpred (https://toolkit.tuebingen.mpg.de/hhpred) (109).

PCR fragments of ORF069 (GenBank accession number ATW59337.1) from phage CL 131 were obtained by amplification of CL 131 wild-type DNA using oligonucleotide primers containing the point base substitutions to generate suitable cloning sites (forward, 5′-GGGGGAGCTAGCATTTTAAACG-3′; reverse, 5′-GCTTGCCCATACTCGAGCTTTGC-3′; restriction sites are underlined). The purified PCR product was cleaved with NheI and XhoI and cloned into the NheI/XhoI digested pET-21b(+) vector (Novagene, USA). This vector was used for the production of gp069 with a noncleavable C terminus hexahistidine tag (gp069_C-his). In order to obtain the gp069 with a noncleavable N terminus histidine tag (gp069_N-his), amplification of gene 069 was performed by using 5′-GGGGGAACTCGAGATTTTAAACG-3′ as the forward primer, and 5′-GTAAAGCTAACTGGATCCCTAGC-3′ as the reverse primer (restriction sites are underlined). The purified PCR product was cleaved with XhoI and BamHI and cloned into the pET-16b vector (Novagene, USA) digested by the adequate restriction endonucleases.

Protein expression was carried out in E. coli strain BL21(DE3) (Novagene, USA). Cell culture was grown at 37°C to an optical density (OD) of 0.5, induced with 0.1 mM isopropyl-β-d-thiogalactopyranoside (IPTG), and incubated overnight at 20°C. Cells were harvested by centrifugation at 4,000 × *g* for 5 min, resuspended in His binding buffer (50 mM sodium phosphate buffer [pH 7.7], 300 mM NaCl, 10 mM imidazole, 0.03% Triton X-100), and disrupted by sonication. Crude extracts were centrifuged at 4°C for 15 min at 21,000 × *g* to remove the debris. Supernatant and pellets were directly analyzed by sodium dodecyl sulfate-polyacrylamide gel electrophoresis (SDS-PAGE). Recombinant His-tagged proteins were purified using a His-Spin Protein Miniprep kit (Zymo Research) according to the manufacturer's recommendations. Concentration of recombinant protein was determined with electrophoresis and by using the method described by Lowry et al. (110).

### Comparison of vB_AphaS-CL131 proteins to metagenomic data sets.

CL 131 proteins were compared against reads from the Baltic Sea (25 metagenomes; NCBI Sequence Read Archive accession number SRP149684), Chesapeake Bay (1 metagenome; retrieved from iMicrobe project CAM_PROJ_CBVIRIO), Lake Matoaka (3 metagenomes; retrieved from iMicrobe project cobian12387), and Lake Michigan (31 metagenomes; retrieved from NCBI BioProject accession number PRJNA248239) metagenomes using tBLASTn (E value of <10^−3^; maximum number of target sequences, 300). The data sets for metagenomic recruitments were selected based on the availability of the metagenomes from the environments in which the appearance of A. flos-aquae was recorded (34). Reads from the Baltic Sea metagenomes with significant matches to CL 131 were further compared to the NCBI nr database (accessed in July 2018) using blastx (E value of <10^−3^) to validate the best significant match.

### Analysis of CRISPR-Cas loci in cyanophage vB_AphaS-CL131 and *A. flos-aquae* genomes.

The CRISPR arrays in cyanophage CL 131 and publicly available A. flos-aquae genomes NIES-81 (NCBI BioProject accession number PRJNA232534), 2012/KM1/D3 (PRJNA257725), MDT14a (PRJNA294801), WA102 (PRJNA294801), and MDT13 (PRJNA294801) were identified and analyzed using CRISPRfinder (http://crispr.i2bc.paris-saclay.fr/Server/) (111) and CRISPRdisco (70) with manual proofreading. The spacers retrieved from A. flos-aquae genomes (in total 561 spacers) were used as queries in the BLASTn search against the CL 131 genome sequence. The multiple sequence alignment of CRISPR-Cas effector proteins was constructed using MAFFT version 7 (112).

### Mass spectrometry identification of virion proteins.

Structural proteins of the CsCl_2_-purified CL 131 particles were analyzed in 15% SDS-PAGE gels using ∼5 µg of total phage proteins. Samples were prepared as described previously (113) and identified *en masse* by liquid chromatography coupled to tandem mass spectrometry (LC-MS/MS). LC-MS/MS analysis was carried out on an EASY-nLC instrument (Thermo Fisher Scientific, Germany) connected to a Velos Pro-Orbitrap Elite hybrid mass spectrometer (Thermo Fisher Scientific, Germany) with a nano-electrospray ion source (Thermo Fisher Scientific, Germany). The LC-MS/MS samples were separated using a two-column setup consisting of a C_18_-A1 trap column followed by a C_18_-A2 analytical column. The linear separation gradient consisted of 5% buffer B for 5 min, 35% buffer B for 60 min, 80% buffer B for 5 min, and 100% buffer B for 10 min at a flow rate of 0.3 µl/min (buffer A consisted of 0.1% trifluoroacetic acid [TFA] in 1% acetonitrile; buffer B consisted of 0.1% TFA acid in 98% acetonitrile). Four microliters of sample was injected per LC-MS/MS run and analyzed. A full MS scan was acquired with a resolution of 60,000 at normal mass range in an Orbitrap analyzer, and the method was set to fragment the 20 most intense precursor ions with collision-induced dissociation (CID) (energy 35) (117). Data were acquired using LTQ Tune software.

Acquired MS2 scans were searched against the CL 131 protein database using the Sequest search algorithms in Thermo Proteome Discoverer. Allowed mass error for the precursor ions was 15 ppm and 0.8 Da for the fragments. A static residue modification parameter was set for carbamidomethyl +57,021 Da (C) of cysteine residue. Methionine oxidation was set as dynamic modification +15,995 Da (M). Only complete tryptic peptides, with a maximum of one missed cleavage, were allowed.

### Determination of cyanophage vB_AphaS-CL131 host range.

Sixty unialgal yet nonaxenic cyanobacterial strains belonging to the genera *Aphanizomenon* and *Dolichospermum* were used for the host range assays (Table S1). Cyanobacteria were cultured in AF-6N_0_ medium (32) under a 14/10-h light-dark cycle and at a light intensity of approximately 120 µmol m^−2^ s^−1^ provided by cool white fluorescent illumination (Philips TL-D 36W/840) at a constant 20°C temperature. For host range assays, cyanophage CL 131 suspensions (50 µl) were inoculated with late-exponential-growth-phase cyanobacterial cultures (200 µl, ∼4 × 10^6^ to 5 × 10^6^ cells ml^−1^) in 96-well plates and incubated for 20 days under the same growth conditions. The control wells received AF-6N_0_ medium (50 µl) instead of CL 131 suspension. An additional 50 µl of growth medium was added to each well (final volume, 300 µl) to prevent nutrient limitation during the incubation period. Cyanobacterial cultures were monitored daily for the presence of well clearance (e.g., until the culture became colorless).

### Accession number(s).

The complete genome sequence of cyanophage vB_AphaS-CL131 has been deposited in the GenBank database under accession number MG209611.

## Supplementary Material

Supplemental file 2

Supplemental file 1
