# Supplementary material for: Genomic Characterization of Cyanophage vB_AphaS-CL131 Infecting Filamentous Diazotrophic Cyanobacterium Aphanizomenon flos-aquae Reveals Novel Insights into Virus-Bacterium Interactions
Source: Appl Environ Microbiol. 2018 Dec 13;85(1):e01311-18. doi: 10.1128/AEM.01311-18 (PMC6293099; doi:10.1128/AEM.01311-18)

Supporting Information

Genomic characterization of cyanophage vB_AphaS-CL131 infecting filamentous diazotrophic cyanobacterium *Aphanizomenon flos-aquae* reveals novel insights into virus-bacterium interactions

Sigitas Šulčius[*1,2], Eugenijus Šimoliūnas[3], Gediminas Alzbutas[1,4,5], Giedrius Gasiūnas[6,7], Vykintas Jauniškis[6,8], Jolita Kuznecova[1], Sini Miettinen[9], Emelie Nillson[2], Rolandas Meškys[3], Elina Roine[10], Ričardas Paškauskas[1] & Karin Holmfeldt[2]

[1]Laboratory of Algology and Microbial Ecology, Nature Research Centre, Akademijos str. 2, LT-08412, Vilnius, Lithuania
[2]Center for Ecology and Evolution in Microbial Model Systems, Department of Biology and Environmental Science, Linnaeus University, SE-39182, Kalmar, Sweden
[3]Department of Molecular Microbiology and Biotechnology, Institute of Biochemistry, Vilnius University, Saulėtekio av. 7, LT-10257, Vilnius, Lithuania
[4]Thermo Fisher Scientific Baltics, Graičiūno str. 8, LT-02241, Vilnius, Lithuania
[5]Faculty of Mathematics and Informatics, Vilnius University, Naugarduko str. 24, LT-03225, Vilnius, Lithuania
[6]Department of Protein-DNA Interactions, Institute of Biotechnology, Vilnius University, Saulėtekio av. 7, LT-10257, Vilnius, Lithuania
[7]CasZyme, Saulėtekio al. 7c, LT-10257, Vilnius, Lithuania
[8]Protein Structure & Function Programme, Structural Molecular Biology Group, Novo Nordisk Foundation Center for Protein Research, Faculty of Health and Medical Sciences, University of Copenhagen, Blegdamsvej 3B, DK-2200 Copenhagen N, Denmark
[9]Proteomics Unit, Institute of Biotechnology, University of Helsinki, Viikinkaari 1, FIN-00014, Helsinki, Finland
[10]Molecular and Integrative Biosciences Research Programme, Molecular Virology Unit, University of Helsinki, Viikinkaari 9, FIN-00014, Helsinki, Finland

*Corresponding author. Mailing address: Nature Research Centre, Akademijos str. 2, LT-08412, Vilnius, Lithuania. Phone: +370-645-91880. E-mail: sigitas.sulcius@gmail.com

List of Supporting Figures

1. SI Figure 1. Dot plot showing the relationship between contig length and number of reads used for the assembly.
2. SI Figure 2. WebLogo-3 representation of the consensus promoter sequences predicted across cyanophage vB_AphaS-CL131 genome.
3. SI Figure 3. TEM micrograph of cyanophage vB_AphaS-CL131, infecting filamentous cyanobacteria *Aphanizomenon flos-aquae* strain 2012/KM1/D3.
4. SI Figure 4. SDS-PAGE of cyanophage vB_AphaS-CL131 virion proteins.
5. SI Figure 6. SDS-PAGE analysis of recombinant gp039_C-his, expressed in E. coli BL21-DE3 cells.
6. SI Figure 6. Multiple sequence alignment of ORF061 and representatives with the Type V-U2 family of CRISPR-Cas effector proteins.
7. SI Figure 7. Figure 8. Hairpin-like secondary structures of V-A system and in cyanophage vB_AphaS-CL131.
8. SI Figure 8. Sequence alignments of ORF067 and *E. coli* HipB proteins.

SI Figure 1. Dot plot showing the relationship between contig length and number of reads used for the assembly. The largest contig (dark blue square) indicate the longest assembled reads, N50 correspond to the minimum contig length needed to cover 50% of the genome and the dotted line indicate the read level used in the subsampling for the genome assembly of cyanophage vB_AphaS-CL131.

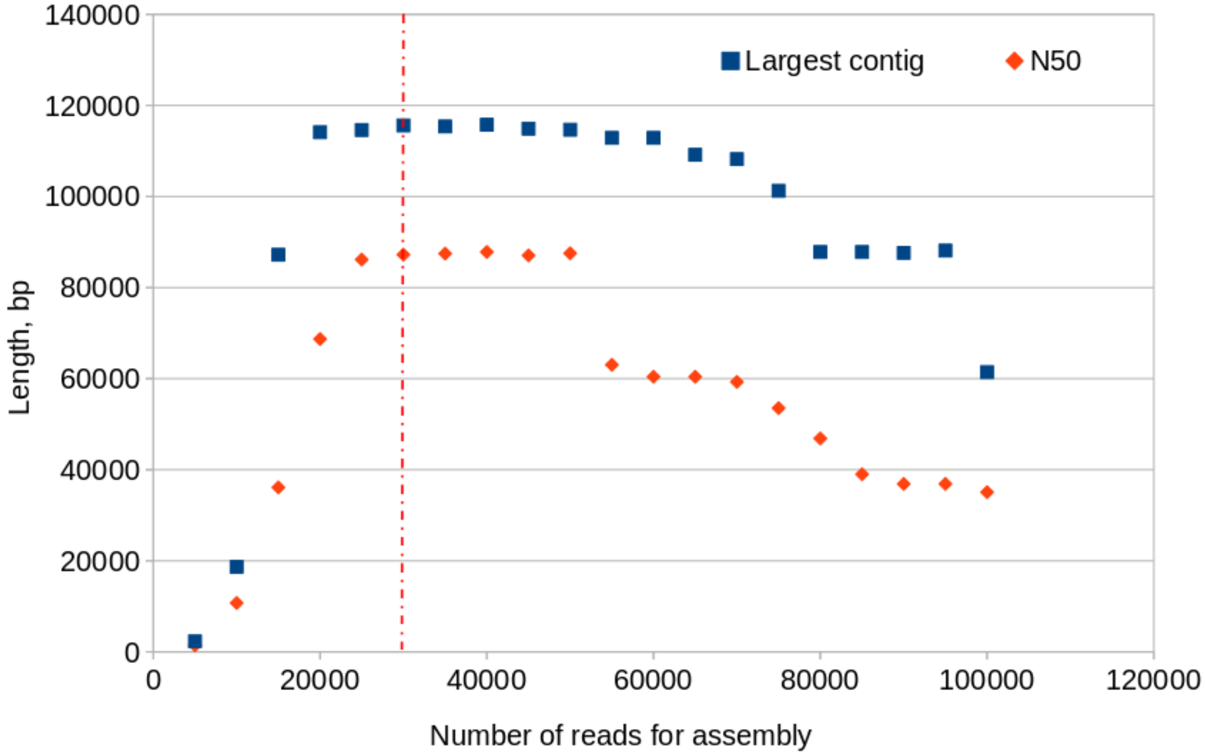

SI Figure 2. WebLogo-3 representation of the consensus promoter sequences predicted across cyanophage vB_AphaS-CL131 genome.

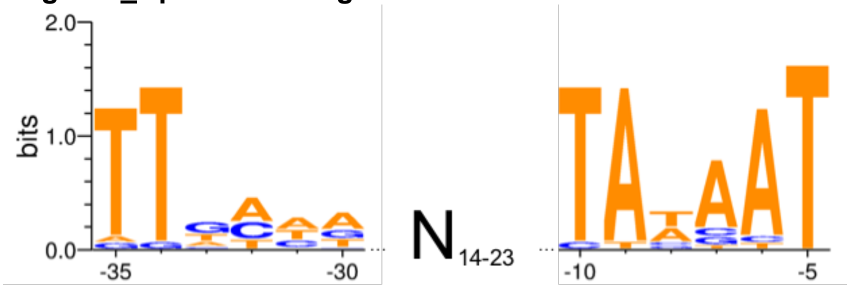

SI Figure 3. TEM micrograph of cyanophage vB_AphaS-CL131, infecting filamentous cyanobacteria *Aphanizomenon flos-aquae* strain 2012/KM1/D3.

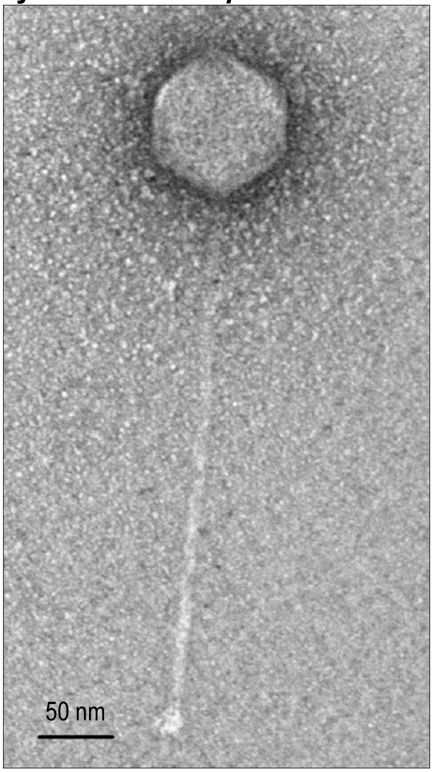

SI Figure 4. SDS-PAGE of cyanophage vB_AphaS-CL131 virion proteins. Lane M - molecular mass marker (Page Ruler pre-stained protein ladder (ThermoFisher)), lane CL131 - cyanophage structural proteins.

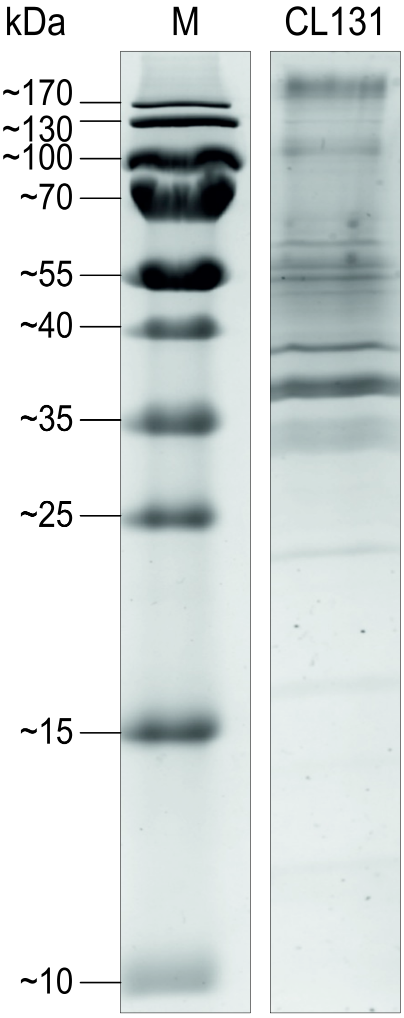

SI Figure 5. SDS-PAGE analysis of recombinant gp039_C-his, expressed in *E. coli* BL21-DE3 cells. Lanes: M – molecular mass marker Page RulerTM prestained Protein Ladder Plus (Thermo Fisher Scientific), 1 – fraction of purified gp039_C-his.

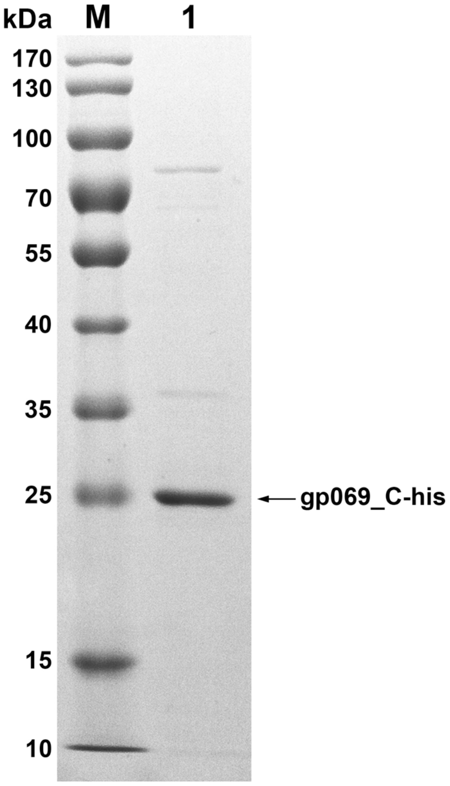

SI Figure 6. Multiple sequence alignment of ORF061 (accession: ATW59329) and representative proteins with the Type V-U2 family of CRISPR-Cas effector proteins (Shmakov et al. 2017). NCBI RefSeq accession IDs are provided for the protein sequences except for the last sequence, where GenBank ID and coordinates are provided. The protein accession numbers correspond to transposases from *Lyngbya aestuarii* (WP_023066538), *Lyngbya* sp. PCC 8106 (WP_009787668), *Microcoleus* sp. PCC 7113 (WP_015183112), *Cyanothece* sp. PCC 8801 (WP_015957291), *Microcystis aeruginosa* (WP_002754940) and *Dolichospermum circinale* AWQC310F (KE384676:18852…20618). Alignments are colored using http://www.bioinformatics.org/sms/multi_align.html server. RuvC catalytic residues indicated by red letters. Identical amino acids are highlighted in black background coloring and groups of amino acids ILMV, FWY, KRH, DE, GAS, C, having ≥80% consensus sequence, in grey.

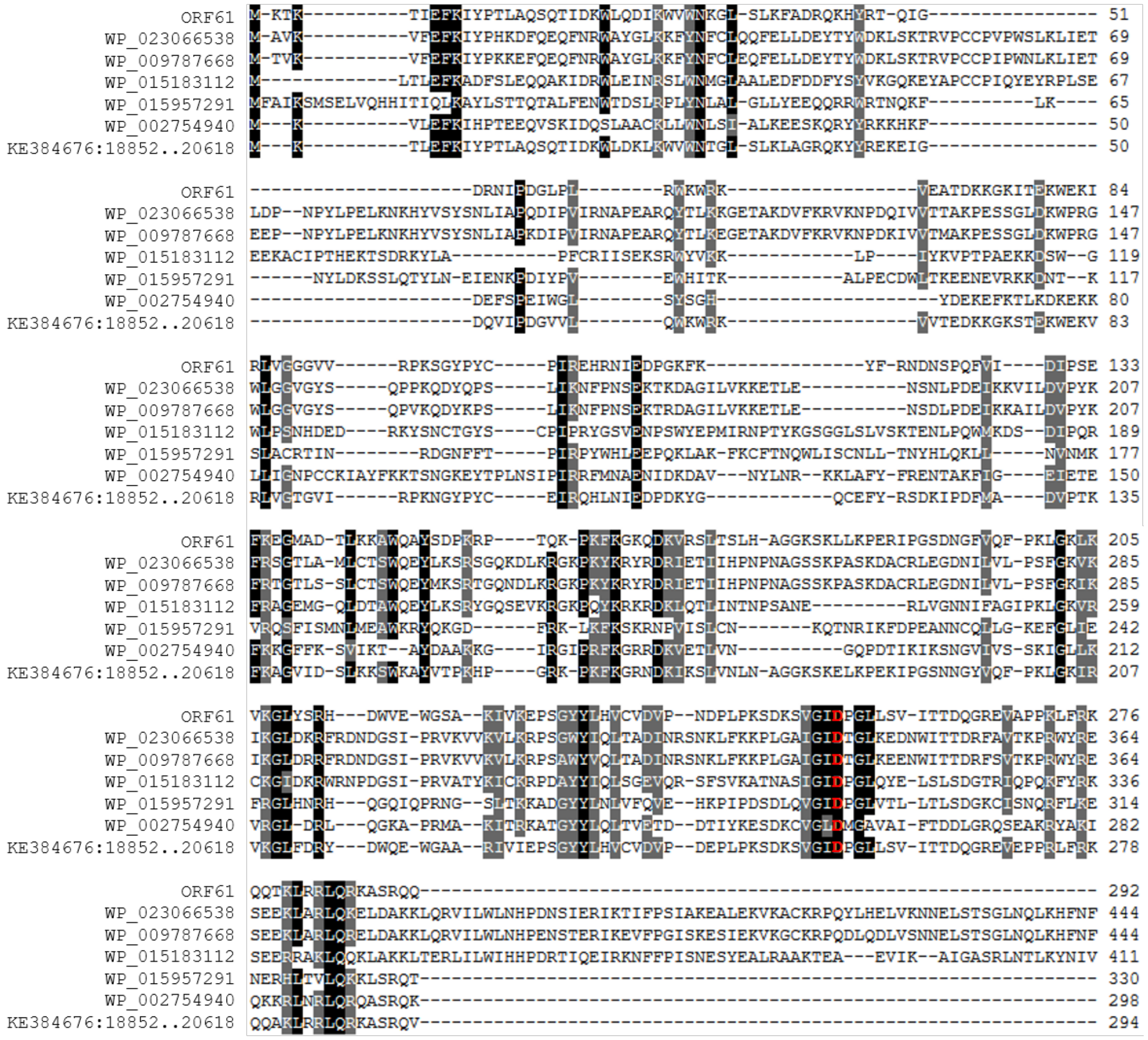

```
               ORF61 ----------------KGGSNQKKTYQKVALQHEKIRRSRNAFNHKLSTKVVREYSGIVMEDLKIQ--NLSRKPKAKKRE 354
        WP_023066538 RDCEKVESCYLFDKLLSASNKEIELAQRIRKLHEKLKRRRRSHNQKQSTWLTRKYSIIRIED-GLQ--KNVGKSKAKVSE 521
        WP_009787668 RDCEKVESCYLFDKLLSVSNKEIELAERIGKLHERNKRRRRSHNQKQSTWLTRKYSIIRIED-GLQ--KNVGKSKAKISE 521
        WP_015183112 PDAPPT----MKDKSPFSGAKQKALEKAIRKLDRKISLQRRNHDHKITTMIVRNYGFIAVED-GLQDEKLRKRTKPKERE 486
        WP_015957291 ----------------PGSKNWEKTKKALAKIHKQTADHRKYYNHKVSTHLVNKYGAIAIEDTKLT--NMNKRPKAEKRE 392
        WP_002754940 ----------------DGSNNQRKTYAKLARVHEKIARQRKGRNAQLAHKITSEYQSVILEDLKLK--NMTAAAKPKERE 360
KE384676:18852..20618 ----------------KGGCNQKKTYRKIALHHEKIRRSRNAFNHKLSTKIVREYSGIVMEDIKIQ--NLNRKPKAKKRE 356

               ORF61 DGNGYEQNGAKRKAGLNKSFADSALGDLISKIETKCKDT-DREFVKVAA---HHTTVDCSNCGAKIKKA--LSQRT---H 425
        WP_023066538 DNRSFERNAQNSRTGMNKSVLDAAIGGFISLVESKSKEW-GRDFKRMKPGKGKAYSQRCPVCHHENKEQKDITNHQD--Y 598
        WP_009787668 DNRSFERNAQNSRAGMNKSVLDAAIGGFISLVEDKSKEW-GRDFKRMKPGKGKAYSQRCPVCHHENKEQKDITNHQD--Y 598
        WP_015183112 DGQGYEQTGAKRKSGLSKSLADASPGRKIAFLKQKADRA-GRVFSQHPA---PYTTKECPVCGSMNEASYNVDDEGNRLY 562
        WP_015957291 DGKGYEHNGAKAKAGLNQSFHDAGLGQLRAFLESKANSYENRHIERVRA---NYTSQKCSRCG-HTDSENRLTQAS---F 465
        WP_002754940 DGDGYKQNGKKRKSGLNKALLDNAIGQLRTFIENKANER-GRKIIRVNP---KHTSQTCPNCG-NIDKANRVSQSK---F 432
KE384676:18852..20618 DGNGYEQNGAKRKAGLNKSFADSALGDLISKIETKCQDT-DREFVKVAA---HYTTVDCSNCGAKIKKA--LSQRT---H 427

               ORF61 RCTECGYED-GRDSNAAKNILIKGQKE-----FKTVYR-------AWAWEHGET-------------------------- 466
        WP_023066538 NCSNCGFTHRSRDVIPGINMILDEFEEQQQKNLLGIET--EEQKIIWDDLSKECRQA-----WRLREKWLS----ENAPG 667
        WP_009787668 NCSNCGFTHRSRDIVPGVNMILDEFEA---------------GDIQWDDLSKEARQA-----WRLREKWLS----ENAPG 654
        WP_015183112 LCIICGWEC-DRDVNSGVNIELAQFGNNPHTVLSANAQRARFANSVWEIAHPEAST------------------------ 617
        WP_015957291 HCLKCGLEM-PADLNAAINIEQTAFGL----------------------------------------------------- 491
        WP_002754940 KCVSCGYEA-HADQNAAANILIRGLRDEFLRAIGSLYKFPVSMIGKYPGLAGEFTPD-----LDANQESIGDAPIENADR 506
KE384676:18852..20618 RCTECGHTE-GRDSNAAKNILLKGKKQ-----LQTVYR-------AWAWEHGETRKPDSECETHCHQEGVQ-APPEDEHS 493

               ORF61 -------------------------------------------------------------------------------- 466
        WP_023066538 G-----------------------------------------------GCQDEVNSDKPSN-TRKSRRRKK--------- 690
        WP_009787668 S-----------------------------------------------GCQDEVTTDKPSN-ARRKRNRKKKT------- 679
        WP_015183112 ---------------------------------------------------------KPR--WKKTEKRKKRK------- 631
        WP_015957291 --------------------------------------------------------------------DKS--------- 494
        WP_002754940 S------TSKQMKQEG-----NRIP--TQSENDSQSLIFLSAPP---QPCEDSHGTNNPKALSDKASKRKSKKSRG-AIP 569
KE384676:18852..20618 SQLTLVRTPAKGTQRGRGDVKTSTPAKTTSKLDTVSDPNLDNKPDTRAPCTSSI---SPNSVENKIPKTKKNKRSAQSSS 570

               ORF61 ------------------ 466
        WP_023066538 ------------------ 690
        WP_009787668 ------------------ 679
        WP_015183112 ------------------ 631
        WP_015957291 ------------------ 494
        WP_002754940 ENPDQLTIWDL-----LD 582
KE384676:18852..20618 ESFTQLTIWDTAGEISFE 588
```

SI Figure 7. Hairpin-like secondary structures of V-A system (left) and in cyanophage vB_AphaS-CL131.

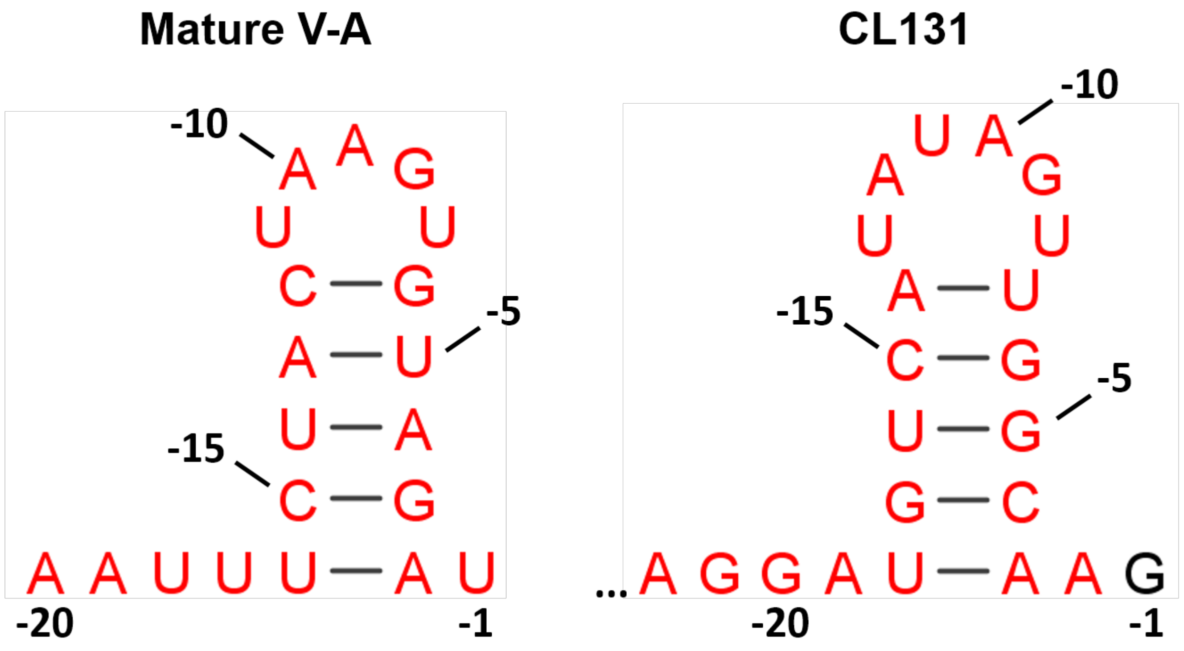

SI Figure 8. Sequence alignments of cyanophage vB_AphaS-CL131 ORF067 (Accession No. ATW59335.1) and *E. coli* HipB proteins (NP_416025.1, P23873.1, KDF68316.1, Q8FHF3.1, KFB93749.1).

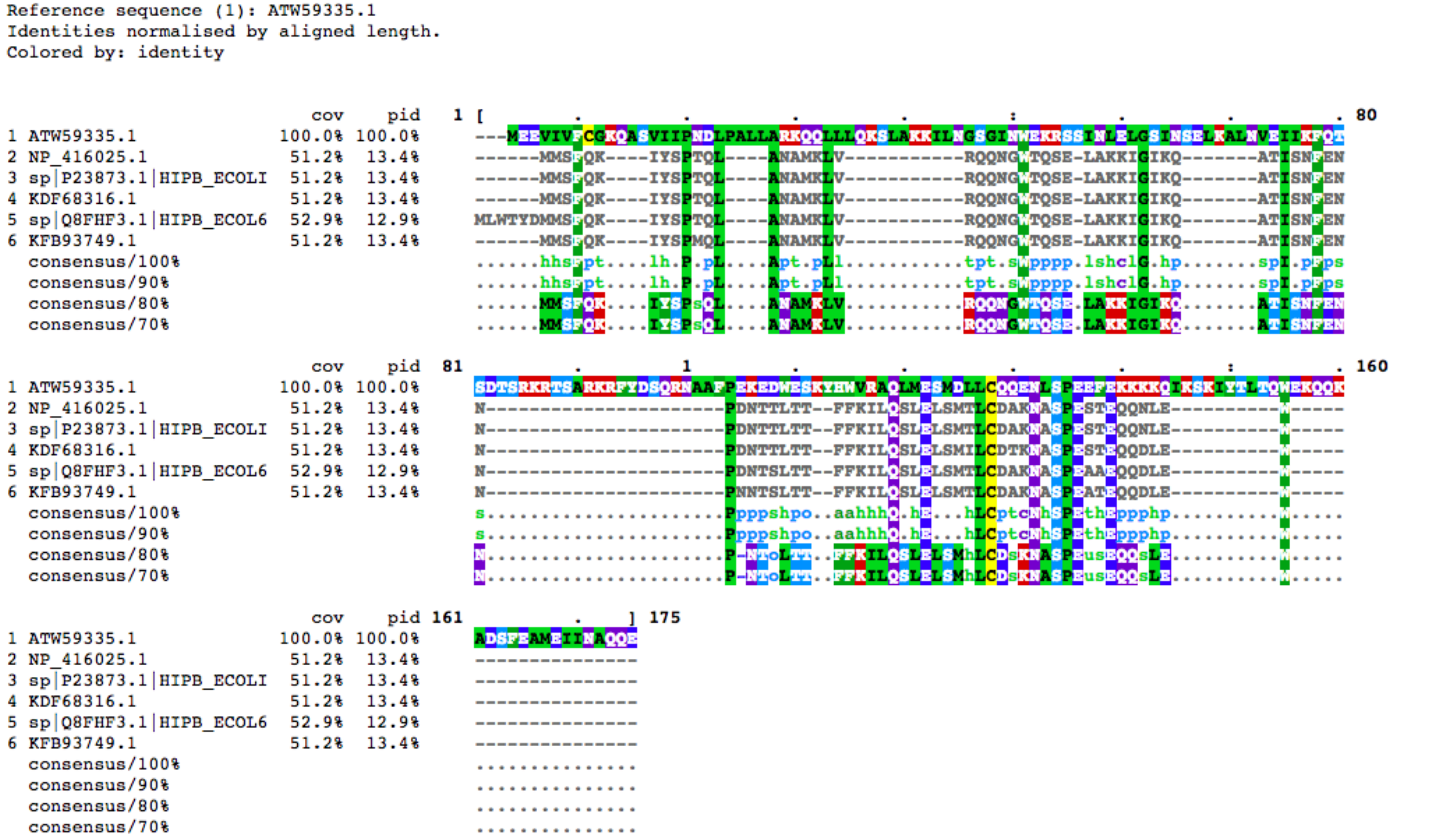

Supplement: Supplemental file 2 [file AEM.01311-18-s0002.pdf]
